# Clinic-Integrated Smartphone App (JomPrEP) to Improve Uptake of HIV Testing and Pre-exposure Prophylaxis Among Men Who Have Sex With Men in Malaysia: Mixed Methods Evaluation of Usability and Acceptability

**DOI:** 10.2196/44468

**Published:** 2023-02-16

**Authors:** Roman Shrestha, Frederick L Altice, Antoine Khati, Iskandar Azwa, Kamal Gautam, Sana Gupta, Patrick Sean Sullivan, Zhao Ni, Adeeba Kamarulzaman, Panyaphon Phiphatkunarnon, Jeffrey A Wickersham

**Affiliations:** 1 Department of Allied Health Sciences University of Connecticut Storrs, CT United States; 2 AIDS Program Yale School of Medicine New Haven, CT United States; 3 Faculty of Medicine University of Malaya Kuala Lumpur Malaysia; 4 Department of Epidemiology Rollins School of Public Health Emory University Atlanta, GA United States; 5 Yale School of Nursing West Haven, CT United States; 6 Love Foundation Bangkok Thailand

**Keywords:** men who have sex with men, mHealth, HIV prevention, pre-exposure prophylaxis, mobile phone, Malaysia, MSM, mobile health, HIV, prevention, usability, acceptability, sexual minority, gay, homosexual

## Abstract

**Background:**

HIV disproportionately affects men who have sex with men (MSM). In Malaysia, where stigma and discrimination toward MSM are high, including in health care settings, mobile health (mHealth) platforms have the potential to open new frontiers in HIV prevention.

**Objective:**

We developed an innovative, clinic-integrated smartphone app called JomPrEP, which provides a virtual platform for Malaysian MSM to engage in HIV prevention services. In collaboration with the local clinics in Malaysia, JomPrEP offers a range of HIV prevention (ie, HIV testing and pre-exposure prophylaxis [PrEP]) and other support services (eg, referral to mental health support) without having to interface face to face with clinicians. This study evaluated the usability and acceptability of JomPrEP to deliver HIV prevention services for MSM in Malaysia.

**Methods:**

In total, 50 PrEP-naive MSM without HIV in Greater Kuala Lumpur, Malaysia, were recruited between March and April 2022. Participants used JomPrEP for a month and completed a postuse survey. The usability of the app and its features were assessed using self-report and objective measures (eg, app analytics, clinic dashboard). Acceptability was evaluated using the System Usability Scale (SUS).

**Results:**

The participants’ mean age was 27.9 (SD 5.3) years. Participants used JomPrEP for an average of 8 (SD 5.0) times during 30 days of testing, with each session lasting an average of 28 (SD 38.9) minutes. Of the 50 participants, 42 (84%) ordered an HIV self-testing (HIVST) kit using the app, of whom 18 (42%) ordered an HIVST more than once. Almost all participants (46/50, 92%) initiated PrEP using the app (same-day PrEP initiation: 30/46, 65%); of these, 16/46 (35%) participants chose PrEP e-consultation via the app (vs in-person consultation). Regarding PrEP dispensing, 18/46 (39%) participants chose to receive their PrEP via mail delivery (vs pharmacy pickup). The app was rated as having high acceptability with a mean score of 73.8 (SD 10.1) on the SUS.

**Conclusions:**

JomPrEP was found to be a highly feasible and acceptable tool for MSM in Malaysia to access HIV prevention services quickly and conveniently. A broader, randomized controlled trial is warranted to evaluate its efficacy on HIV prevention outcomes among MSM in Malaysia.

**Trial Registration:**

ClinicalTrials.gov NCT05052411; https://clinicaltrials.gov/ct2/show/NCT05052411

**International Registered Report Identifier (IRRID):**

RR2-10.2196/43318

## Introduction

Men who have sex with men (MSM) are disproportionately affected by HIV in Malaysia and accounted for 63% of new HIV diagnoses in 2021, a proportion that has been increasing over the past decade [1,2]. This pattern requires implementation of more effective HIV prevention in MSM, yet in Malaysia, as in many low- and middle-income countries (LMICs), MSM often do not adequately access evidence-based HIV prevention (and treatment). Gaps in prevention and treatment are due, in part, to high levels of social stigma and discrimination against MSM. In Malaysia, these factors are heightened further because same-sex sexual behaviors are criminalized [2-4]. Other factors also contribute to low uptake of services, including sexual networks that have evolved through social networking apps. Transmission potential is heightened by behavioral or biological factors, including condomless sex, multiple concurrent sexual partners, substance use, and mental health problems (eg, depression, anxiety) that act synergistically to increase the HIV risk in this group [5-12].

Routine HIV testing and expanded use of pre-exposure prophylaxis (PrEP) would drastically reduce the population-level burden of HIV [13-16]. Uptake of these evidence-based tools, however, is suboptimal among Malaysian MSM. For example, recent data suggest that 55% of MSM reported not having tested for HIV in the past 6 months, and approximately 30% reported they had never been tested [17-19]. Additionally, only 18.3% of MSM with indications for PrEP reported ever using it, despite high awareness and willingness to use PrEP [20,21]. This low uptake is partly explained by the need to maintain meaningful engagement with the health care system to access these services. Yet, it is often difficult for MSM in Malaysia to find culturally appropriate health care services due to known barriers, such as discomfort and distrust associated with disclosing sexual behavior to providers for fear of ramifications [22]. As such, there is a need for innovative strategies to improve access to HIV prevention services for Malaysian MSM.

Mobile health (mHealth), particularly smartphone apps, holds great promise for HIV prevention [18,23-26], especially when linked to accessible HIV testing and PrEP. App-based interventions can help overcome multilevel barriers, given their ability to anonymously reach and engage populations that are disenfranchised from existing prevention efforts and offer “real-time” delivery and rapid scalability of programs at relatively low implementation costs. In Malaysia, smartphone use among MSM is nearly universal, and MSM report a strong preference for app-based HIV prevention programs [18,19]. Although app-based interventions are evolving and promote the HIV prevention continuum, most, if not all, are limited to high-income countries and none provide comprehensive HIV prevention services [27]. Furthermore, many of these emerging apps deploy an online-to-offline (O2O) strategy [28,29], where clients eventually must be seen in person. In these cases, the anonymity afforded through the online experience ends during the clinical encounter where individuals must be linked to their testing results and medication prescriptions. An important innovation in the traditional O2O and clinic-based models would be to keep the entire care process in the virtual space (online). However, such strategies have yet to be developed and assessed.

To address this unmet need, we developed JomPrEP (where “Jom” means “let us” in Bahasa Malaysia), a clinic-integrated smartphone app, designed to provide HIV prevention services for MSM in Malaysia. The development of JomPrEP has been described previously [30]. In brief, it is adapted from HealthMindr, an app previously demonstrated to increase HIV testing and PrEP uptake among MSM in the United States [25]. In collaboration with local Malaysian clinics, JomPrEP offers a virtual platform for Malaysian MSM to access a range of HIV prevention (ie, HIV testing and PrEP) and other support services (eg, referral to mental health support) without having to interface face to face with clinicians. It includes several on-demand features, including scheduling and managing appointments in person or through e-consultation, communicating with the clinical team (ie, chat), home-based testing, accessing test results, ordering health products, discrete door-to-door delivery, timely notifications, a points-based reward system for completing activities within the app, and a multimedia resource center. Here, we report findings from beta testing of the recently developed JomPrEP app to evaluate its usability and acceptability.

## Methods

### Study Design and Settings

We conducted beta testing of JomPrEP to assess its usability and acceptability among MSM living in the Greater Kuala Lumpur region, Malaysia. Beta testing of an app helps to identify any final areas for improvement [31]. We hypothesized that beta testing for 30 days of observation (N=50) would allow us to evaluate the app's design, functionality, and usability. The sample size of 50 was determined based on the pragmatics of recruitment and the need to examine feasibility [32-34].

We partnered with the Centre of Excellence for Research in AIDS (CERiA) at the University of Malaya, Kuala Lumpur, Malaysia, to conduct this study. Working closely with several other local and international institutions, CERiA conducts innovative and interdisciplinary research that combines epidemiological, biomedical, and sociobehavioral approaches, focusing on the implementation of HIV prevention and treatment. As part of JomPrEP integration with existing clinics, we partnered with 2 local clinics—the Red Clinic (private clinic) and the Community Health Care Clinic (nongovernmental organization [NGO]-based clinic)—to provide clinical services (eg, HIV testing, sexually transmitted infection [STI] testing, PrEP services) virtually via the app.

### Study Participants and Recruitment

Eligibility criteria included (1) being 18 years or older; (2) identifying as a cis-gender man; (3) self-reporting an HIV-negative or HIV status unknown at screening; (4) not having used PrEP previously (ie, PrEP naive); (5) self-reporting evidence of being at risk for HIV acquisition, as defined by the World Health Organization PrEP clinical guidelines [35]; (6) owning a smartphone; and (7) currently residing in the Greater Kuala Lumpur region.

In total, 50 participants were recruited between March and April 2022 using in-person and online recruitment strategies. For in-person recruitment, flyers were distributed to potential participants as well as posted at local partner organizations (eg, clinics, lesbian, gay, bisexual, transgender [LGBT]–friendly community-based organizations). Additionally, we used various general and MSM-specific social media platforms as venues for participant recruitment. These included placing advertisements in geosocial networking (GSN) apps popular among MSM in Malaysia (ie, Hornet) as well as posting study flyers on Malaysian MSM–focused Facebook pages. Interested individuals who clicked on an advertisement were directed to the study website [36], where they were presented with a brief description of the study and web-based screening.

### Procedures

After meeting enrollment criteria, eligible participants were asked to provide electronic informed consent for study participation, followed by undergoing a baseline assessment. Study staff then assisted enrolled participants with downloading JomPrEP and provided them with brief instructions on the purpose of the app and an overview of how to use it. To restrict access to JomPrEP to the study participants, participants were provided with a single-use registration code needed to gain access to the app. Upon downloading the app, participants were asked to complete an onboarding process, which included creating log-in credentials. They were then redirected to the JomPrEP landing screen (home screen), which contains several icons representing key app functions (Table 1). Screenshots of the app are available in Multimedia Appendix 1. Participants were requested to keep and use the app for 30 days and encouraged to use all app features. On day 30, participants completed a posttest survey and were asked to provide a synthesis of issues regarding the app (ie, exit interviews). For exit interviews, 20 (40%) participants were randomly sampled and interviews were conducted until data saturation was reached. The 1-on-1 sessions were conducted online via licensed videoconferencing software. Participants were given the choice of turning on or off their cameras and were asked to use a pseudonym/nickname.

Participants received point-based rewards (known as JomPrEP points [JPP]) for completing specific activities or meeting milestones via the app (eg, 100 JPP for baseline and follow-up assessment each, 50 for completing app onboarding, 20 for an HIVST in-app order, 50 for completing a lab test, 50 for completing an e-consultation for PrEP, 30 for an in-app PrEP order, 50 for optimal PrEP adherence; maximum points that could be earned: 740 or US $18.50). Participants were allowed to redeem points for cash at any point during the study period (10 JPP=RM 1, or US $0.24).

**Table 1:** Main features of JomPrEP.

Features	Description
Customizable Home page	Visual presentation using avatars and pseudonyms
HIVST^a^	Allows users to order an HIVST kit (Orasure) Allows users to upload HIVST results for verification purposes to facilitate posttest linkage to HIV prevention or treatment services Includes multimedia (ie, text-, picture-, and video-based) content on how to use the HIVST kit and interpret the result
PrEP^b^ Express	Provides users with a fast and convenient way to start PrEP (and state on it) Includes a sequential pathway for the users to get on PrEP: HIV risk assessment (provides tailored recommendations based on the user’s response); choose preferred clinic (allows users to choose between the participating clinics for PrEP); choose PrEP type (allows users to choose between different PrEP prescription modalities, ie, same day^c^ vs traditional^d^); schedule appointments (allows users to choose their preferred date and time for phlebotomy and e-consultation^e^); PrEP medication delivery (allows users to choose their preferred method of getting PrEP: pickup at the pharmacy vs discrete door-to-door delivery)
Orders	Allows users to monitor past and current orders (HIVST kit, PrEP) Allows users to track their current orders in real time (via application programming interface [API] integration of courier tracking) Provides notifications on the status of their orders
Labs	Allows users to view their lab test results Allows users to receive timely notifications about new lab results
Appointments	Allows users to view details about past and future appointments and make any necessary changes (eg, reschedule, cancel) Allows users to meet with a doctor virtually (ie, e-consultation)
Mental Health	Allows users to self-screen for depression and receive results Provides users with community resources and support services (ie, a list of mental health service providers) Provides users with a personalized referral letter to mental health services to facilitate rapid linkage^f^ Allows users to keep track of previous mental health–screening results and access referral letters
MedManager	Allows users to set and receive personalized medication reminders Provides users with reminders to refill their prescriptions Allows users to view visual reports of their medication adherence
MoodTracker	Allows users to keep track of their mood daily Provides users with a visual display of their mood over time
Messages (ie, chat)	Allows users to send and receive nonurgent medical questions to the clinic and research staff
Resources	Provides users with multimedia (text-, picture-, video-based) content of an array of relevant information (eg, HIV, PrEP, substance use, mental health)
News	Provides users with the latest health news updated regularly
Reward points	Allows users to accumulate points for completing activities in the app Allow users to track and redeem points for cash or in-app purchases
JomPrEP Clinic Dashboard	Includes a web-based dashboard for the clinics affiliated with the JomPrEP app Allows staff members of the affiliated clinics (ie, doctors, nurses, pharmacists, front-desk staff) to access the dashboard to facilitate patient care for JomPrEP app users In the absence of an electronic health record (EHR) in the local setting, functions more like the EHR system

^a^HIVST: HIV self-testing.

^b^PrEP: pre-exposure prophylaxis.

^c^Receive PrEP at the first doctor visit (no need to wait for lab results).

^d^Get PrEP after lab results are complete (need a follow-up doctor visit to review lab results).

^e^Applies only to those who choose traditional PrEP.

^f^Includes a referral letter for mental health.

### Ethical Considerations

The Institutional Review Board at the University of Connecticut approved this study (H22-0049), with an institutional reliance agreement with the University of Malaya. Eligible participants provided electronic informed consent for study participation.

### Assessments

#### Participant Characteristics

All assessments (ie, baseline and follow-up) were conducted virtually and self-administered using Qualtrics. We collected participant demographic and baseline characteristics, including age, ethnicity, educational status, relationship status, income, housing status, depressive symptoms [37], substance use and sexual history, HIV/STI-testing practices, and past use of PrEP and postexposure prophylaxis (PEP).

#### JomPrEP App Evaluation

After 30 days of use, participants were asked to assess the app’s features, usability, design, content, and functionality using 2 Likert scales. JomPrEP acceptability was assessed using the System Usability Scale (SUS) [38], a validated measure that assesses the subjective usability of an app. Scores range from 0 to 100, with scores of ≥50 indicating that the app is acceptable [38]. We also collected app analytics, such as the number of log-ins, session duration, pages visited, and frequency and duration of use of app components, to determine usability. Additionally, data on the uptake of HIV testing and PrEP, mental health screening and referral to mental health support services, and use of the HIVST kit for those who placed in-app orders were extracted from the web-based JomPrEP Clinic Dashboard.

Finally, we conducted exit interviews with 20 (40%) participants to obtain feedback on app functionality, technical performance, errors and software bugs encountered, overall experience using the app, feedback for further refinement, and subjective impact of the app on HIV testing and PrEP uptake. One-on-one interviews were conducted by research staff virtually using videoconferencing technology. Interviews were recorded and transcribed for analysis.

### Analytical Plan

All quantitative data were managed and analyzed using IBM SPSS Statistics version 28. Means for continuous variables and frequencies for categorical variables were calculated to describe the participants at baseline. App usability and acceptability were based on descriptive statistics from the app analytics and acceptability measure. For example, evaluation responses are reported as the percentage of users who completed the posttest survey. SUS results are reported as an aggregate score, with a score of ≥50 indicating that the app is acceptable [39,40]; the percentage of participants with scores ≥50 is also reported. Descriptive statistics of app analytics were used to examine app engagement and are reported as the mean with the range for time and action measurements. For qualitative data, all the exit interviews were audio-recorded, transcribed, and analyzed. The comments and issues were grouped and categorized according to common themes relative to specific app functions by 3 coders (including 2 senior coders) and agreed upon by all authors. Dedoose version 9.0.54 was used throughout to assist in data management and analysis.

## Results

### Participant Characteristics

The mean age of the 50 participants was 27.9 years (range 21-45 years), with most being single (36/50, 72%), Malay (26/50, 52%), university graduates (34/50, 68%) and living in a house/apartment with other people (36/50, 72%). Almost all participants reported having been tested for HIV (49/50, 98%), and 39/50 (78%) participants had done so in the past 6 months. Of all 50 participants, 26 (52%) reported using HIVST and only 5 (10%) had used PrEP previously. Regarding sexual behaviors in the past 6 months, 47/50 (94%) participants reported anal sex with another man, while only 16/50 (32%) participants reported consistent condom use, 4/50 (8%) reported having engaged in sexualized drug use, and 9/50 (18%) reported having engaged in group sex (Table 2).

**Table 2:** Characteristics of participants (N=50).

Variables		Frequency
Age (years), mean (SD)		27.9 (5.3)
**Ethnicity (Malaya), n (%)**		
	No	24 (48)
	Yes	26 (52)
**University graduate^a^, n (%)**		
	No	16 (32)
	Yes	34 (68)
**Relationship status, n (%)**		
	Single	36 (72)
	Partner	14 (28)
Monthly income (RM/US $), mean (SD)		3553.40 (2985.90)/837.97 (704.14)
**Living status, n (%)**		
	Alone	14 (28)
	Living with others	36 (72)
**Tested for HIV (past 6 months), n (%)**		
	No	11 (22)
	Yes	39 (78)
**Ever used an HIVST^b^ kit, n (%)**		
	No	24 (48)
	Yes	26 (52)
**Previously diagnosed with STI^c^, n (%)**		
	No	27 (54)
	Yes	23 (46)
**Ever used PrEP^d^, n (%)**		
	No	45 (90)
	Yes	5 (10)
**Ever used PEP^e^, n (%)**		
	No	46 (92)
	Yes	4 (8)
**Perceived HIV risk, n (%)**		
	None	6 (12)
	Low	25 (50)
	Moderate	15 (30)
	High	4 (8)
**Ever injected drugs, n (%)**		
	No	49 (98)
	Yes	1 (2)
**Engaged in anal sex (past 6 months), n (%)**		
	No	3 (6)
	Yes	47 (94)
**HIV serodiscordant relationship (past 6 months), n (%)**		
	No	47 (94)
	Yes	3 (6)
**Consistent condom use (past 6 months), n (%)**		
	No	34 (68)
	Yes	16 (32)
**Engaged in group sex (past 6 months), n (%)**		
	No	41 (82)
	Yes	9 (18)
**Engaged in sexualized drug use^f^ (past 6 months), n (%)**		
	No	46 (92)
	Yes	4 (8)

^a^Includes college, university, and professional degrees.

^a^HIVST: HIV self-testing.

^c^STI: sexually transmitted infections (eg, gonorrhea, chlamydia, syphilis).

^d^PrEP: preexposure prophylaxis.

^e^PEP: postexposure prophylaxis.

^f^Use of psychoactive substances (eg, amphetamines, 3,4-methyl​ene dioxy​methamphetamine [MDMA]) before or during sexual activity.

### Uptake of HIV Prevention Services

During the 30-day beta-testing phase of JomPrEP, 42/50 (84%) participants ordered an HIVST kit using the app. Almost all (46/50, 92%) participants used the app to get on PrEP. Specifically, 30/46 (65%) participants chose same-day PrEP versus traditional PrEP, and the majority of them picked up the PrEP medication at the pharmacy (28/46, 61%). Additionally, 44/50 (88%) participants used the online assessment tool to screen for depression, and 39/44 (89%) of them met the criteria for moderate-to-severe depressive symptoms [37] and were provided with a referral letter (Table 3).

**Table 3:** Participants’ uptake of HIV testing and PrEP^a^ services using JomPrEP (N=50).

Service usage		Frequency
**HIV testing**		
	Ordered HIVST^b^ kit	42 (84)
	Verified HIVST results^c^	40 (95)
**Linked to PrEP services (n=46, 92%)**		
	Traditional^d^ PrEP delivery	16 (35)
	Same-day^e^ PrEP delivery	30 (65)
	Completed phlebotomy	46 (100)
	Completed e-consultation^f^	16 (35)
	Completed in-person consultation	30 (65)
	Picked-up PrEP medication at pharmacy	28 (61)
	PrEP medication delivered at home	18 (39)
Mental health screening		44 (88)

^a^PrEP: preexposure prophylaxis.

^b^HIVST: HIV self-testing.

^c^HIVST result verified by providing an image of the result via the app.

^d^Receive PrEP after lab results are complete (need a follow-up doctor visit to review lab results).

^e^Receive PrEP at the first doctor visit (no need to wait for lab results).

^f^Applies only to those who choose same-day PrEP.

### JomPrEP App Evaluation

During the beta-testing phase, 29/50 (58%) participants were Android users, while the remainder (21/50, 42%) were iOS users. Usability measures by participants included app use, with an average of 8 (SD 5.0, range 2-18) unique visits over 30 days, with an average duration of 28 (SD 38.9) minutes per session. The app had a mean of 34.9 (SD 14.7) daily users, with 939.3 (SD 597.9) daily page views, 63.4 (SD 28.5) daily sessions on average, and consistent returning visits (eg, >10: 29/50, 58%; 6-10: 22/50, 44%).

The mean acceptability score was 73.8 (SD 10.1) on the SUS, well above the minimum criteria (≥50) set for the acceptability of the app [39,40], with all participants reporting acceptability scores of >50. Almost all participants reported that they were satisfied with JomPrEP (46/50, 92%) and that the app was useful in addressing their HIV prevention needs (49/50, 98%); see Table 4.

When participants were asked about future app use, most said they were likely to continue using the app as part of their HIV prevention plan (42/50, 84%), would download the app if publicly available (43/50, 86%), and would recommend the app to their friends or colleagues (50/50, 100%). Most participants felt confident in in-app security (43/50, 86%), including autologout after 5 minutes of inactivity (44/50, 88%), an email and password log-in (42/50, 84%), a 4-digit personal identification number (36/50, 72%), and the app name and icon not associated with HIV (32/50, 64%); see Table 5.

Participants found JomPrEP to be easy to use and felt confident that they would be able to learn how to use it quickly and without technical assistance (Table 6).

**Table 4:** Participants’ rating of satisfaction with using JomPrEP features (N=50).

Activity (“How satisfied are you with the following features of the JomPrEP app?”)	Participants, n (%)^a^
Ordering an HIVST^b^ kit	47 (94)
Ordering PrEP^c^ medication	46 (92)
Reward system (earning and redeeming points)	43 (86)
Completing the mental health screener	42 (84)
Chat with clinical or research staff	42 (84)
Booking appointments (blood draw, consultation with doctor)	42 (84)
Keeping track of upcoming and past appointments	42 (84)
Tracking order status (ie, HIVST, PrEP)	40 (80)
MoodTracker (track mood daily)	40 (80)
Online consultation with the doctor (e-consultation)	40 (80)
Reviewing laboratory test results	38 (76)
Resources/News Center	36 (72)
Notifications from the app	36 (72)
MedManager (receive medication reminders, track medication use)	30 (60)

^a^Very satisfied and extremely satisfied (not included: not at all satisfied, slightly satisfied, moderately satisfied).

^b^HIVST: HIV self-testing.

^c^PrEP: preexposure prophylaxis.

**Table 5:** Participants’ rating of the usefulness of JomPrEP features (report their use; N=50).

Activity (“How much do you agree that the use of the JomPrEP app…”)	Participants, n (%)^a^
Assisted in getting tested for HIV	50 (100)
Assisted in getting started on PrEP^b^	50 (100)
Made access to medical records easier (ie, test results, appointments)	49 (98)
Made access to HIV testing much easier	49 (98)
Helped to understand the risk of getting HIV	46 (92)
Motivated to get on PrEP	46 (92)
Motivated to get tested for STIs^c^	46 (92)
Helped to understand whether PrEP would be a good fit	46 (92)
Helped to get in touch with the clinic staff (via chat messages)	44 (88)
Helped to get the latest information about HIV	43 (86)
Helped to understand mental health needs	42 (84)
Made access to PrEP much easier	40 (80)

^a^Agree and strongly agree (not included: strongly disagree, disagree, neither agree nor disagree).

^b^PrEP: preexposure prophylaxis.

^c^STI: sexually transmitted infection.

**Table 6:** Participants’ rating of the level of difficulty of JomPrEP app features (N=50).

Activity (“How easy or hard was it to do the following tasks on the JomPrEP app?”)	Participants, n (%)^a^
Ordering an HIVST^b^ kit	49 (98)
Ordering PrEP^c^ medication	47 (94)
Booking appointments (blood draw, consultation with doctor)	46 (92)
Reward system (earning and redeeming points)	44 (88)
Completing a mental health screener	43 (86)
Customizing profile page (eg, avatar, password, address, name)	43 (86)
Tracking the order status (ie, HIVST, PrEP)	42 (84)
Creating an account (onboarding process)	40 (80)
Reviewing laboratory test results	40 (80)
MoodTracker (track mood daily)	40 (80)
Chat with clinical or research staff	38 (76)
Find relevant information about HIV prevention	35 (70)
Online consultation with a doctor (e-consultation)	33 (66)
MedManager (receive medication reminders, track medication use)	25 (50)

^a^Very easy (not included: very difficult, difficult, neutral).

^b^HIVST: HIV self-testing.

^c^PrEP: preexposure prophylaxis.

### Exit Interviews

In follow-up exit interviews (n=20, 40%), participants indicated a high level of acceptability for the content, interface, and features of JomPrEP. Participants found the app to be user friendly, easy to navigate, and with a good layout. Participants also appreciated the ability to earn reward points for using specific app features, facilitating user engagement and retention.

…it's straightforward, it's user friendly, it's easy to use…I think the critical part for me is actually the ease of use of the app.

Because of convenience. It’s like having a mini-doctor. It’s much easier for you to get tested, instead of going to a clinic and stuff.

The point and rewards system are very interesting and attractive.

Participants noted that ordering HIVST kits via the app was straightforward and that the multimedia instructions helped them use HIVST kits and interpret test results.

I tried self-test kits from other sources, comparing the experience using this and also ordering elsewhere, I think JomPrEP was very prompt, the delivery was, I think, the next day and self-test kit was easy to use.

The feature I used most in the app is the one that allowed me to order a self-testing kit. It’s super convenient because first, order placement is very easy to do and the second one because the delivery is very fast…And they have very clear instructions on how to do it and get the result. Instructions to do the screening at home are very clear. And to upload the result is easy as well.

Furthermore, participants endorsed that the app helped them initiate PrEP use and maintain optimal adherence by facilitating a safe and stigma-free virtual platform to access PrEP services. Participants commented on the relevant information presented in the app, and many noted that they “didn’t know anything about [PrEP] until [they] used the app.”

There’s a lot of information that makes me want to take PrEP more, because of the useful information and why I need to take the PrEP. This app is very like a one-stop center to take the PrEP…inside, you can book a consultation, view your result and you can directly order the PrEP. So, this helped me more easily to get the PrEP.

I have been thinking of getting PrEP but didn’t know much about it (process, the cost, etc). The app makes everything more transparent. And when you take it, there’s a reminder every day so you can set up the clock, so you don’t forget.

When I first came here, I am not originally from here, I was trying to find PrEP, and I had a hard time finding it. When I used this app, it made things much easier. I don’t need to worry if the clinic is judgmental.

Participants also provided suggestions to improve the app and specific feedback on additional resources and features that they found interesting and helpful. For example, participants suggested that the app include an option to make an appointment with a mental health counselors and support groups and the ability to connect with other JomPrEP users through private messaging or discussion forums. Participants shared occasional issues with lagging app response time, difficulty setting up reminder notifications, and missing notifications. A few participants indicated that the test result feature of the app was a little challenging to use and required multiple clicks to view the results. A few participants also noted that some of the information in the app is repetitive or is not updated frequently. One participant recommended that the app allow the users to make the app more discreet (eg, the ability to change app icons).

Participants indicated that they would continue to use the JomPrEP app after the final version is released to the public.

Yes, definitely. I would use it because it's easier to put my appointment and view my lab results. I don't have to have it in a hardcopy form, easily accessible to my smartphone, and I could easily order my HIV self-testing kit as well.

I will continue to use it. And I think the JomPrEP app is very useful for me in terms of ordering the PrEP and booking e-consultations.

## Discussion

### Principal Findings

Using innovative tools, such as mHealth, in public health programming and the health care system can help bridge gaps in the adoption of needed health and prevention services, particularly among underserved populations [41-44]. In this study, we sought to investigate the usability and acceptability of JomPrEP, a clinic-integrated smartphone app, as an additional platform to promote routine HIV testing and PrEP uptake among MSM in Malaysia. Our findings demonstrated that Malaysian MSM will use a smartphone app to virtually access HIV prevention services and that such an app is acceptable to this at-risk group, as indicated by the participants’ empiric use of the app.

### Comparison With Prior Work

Prior studies have demonstrated several apps for HIV prevention and treatment efforts to be promising and cost-effective strategies to reach and engage stigmatized and hidden populations, such as MSM [25,45-47]. In Malaysia, the use of mobile technology over the past decades has grown markedly, particularly among MSM, with a mobile phone penetration rate of 97.5% and an internet penetration rate of 71.1% [18,19,48]. Importantly, our beta testing of JomPrEP revealed that an overwhelming majority of men used the app to receive HIV prevention services: ordering an HIVST kit (84%) and getting on PrEP (94%). Participants reported that they were satisfied and comfortable using JomPrEP and would recommend it to friends or colleagues. These findings indicate the potential utility of JomPrEP for Malaysian MSM to promote HIV prevention services.

One of the key innovations on JomPrEP includes incorporating on-demand features, such as home-based HIVST, e-consultations, and discrete door-to-door delivery, to provide a scalable model for remote HIV prevention services delivery in the LMIC setting. Although the users would still be required to visit a laboratory for clinical testing, this would not require them to be face-to-face with their clinicians. Moreover, the platform allows users to self-assess their HIV risk, consult online with clinicians from the participant clinics, and have their medication delivered to their preferred location, thus minimizing the need for in-person interactions with the clinician. This represents a significant and much-needed innovation over traditional clinic-based and O2O models of HIV service delivery to keep at-risk individuals wedded into the virtual clinical ecosystem and boost the uptake of clinical services [28,29]. This is particularly important in LMIC settings, such as Malaysia, as the virtual platform allows users to bypass barriers to care for marginalized populations and feel safer and less vulnerable to potential legal or social harm (eg, by reducing face-to-face interactions with providers).

Prior research has documented low user retention and a lack of sustained use after adoption as key challenges to the effectiveness of existing app-based interventions [49]. Obtaining high engagement and retention is necessary to maintain the integrity and long-term sustainability of effective mHealth interventions [50]. Strategies to integrate other features that do not include individual input, such as passive data collection using inputs from their smartphones or unobtrusive wearable devices, may strengthen the features of the app.

Results from our beta testing, however, revealed that MSM were actively engaged in the app and that retention was excellent through the beta testing. Although it is possible that the perfect retention rate could be because of the shorter follow-up time, it is likely that the incorporation of additional components, such as the ability to customize profiles, personalized messages, and gaming elements (ie, the ability to “level up,” earn and redeem points), may have allowed for enhanced user engagement. In recent years, the utility of gamification features (eg, challenges, tasks, rewards, badges, leaderboards) in nontraditional gaming contexts has increased significantly, thus providing opportunities for greater user engagement in mHealth interventions [51,52]. As confirmed in the exit interviews, the overall high engagement and usage of key features of JomPrEP suggest that an app-based intervention, such as JomPrEP, has a high degree of feasibility to ensure equitable access to HIV testing and PrEP services for MSM in Malaysia.

Although there was consensus on the usability and acceptability of JomPrEP, with no significant differences between different subgroups of Malaysian MSM, the app would benefit from continued refinement to address some of the shortcomings identified by men. For example, most participants who completed screening for depressive symptoms (88%) received referral letters to seek care offline (ie, outside the app). Given the focus of JomPrEP to offer holistic HIV prevention services within the online ecosystem, it would be important for the app to incorporate online consultation with mental health counselors and linkage to support groups via the app. Additionally, as part of the continued effort to ensure the safety and security of users, it would be important that JomPrEP incorporate added security measures, including 2-factor authentication and a discreet app icon (DAI). The availability of a DAI allows users to replace the default JomPrEP app log on their phone with another symbol (of their choice). This will help protect users when there is a possibility that someone may accidentally look at users’ phones and recognize that they have an app that might link them to the HIV or lesbian, gay, bisexual, transgender, queer, and others (LGBTQ+) community. A study conducted with MSM of Malaysia also highlighted the importance of privacy and confidentiality features in the mobile apps targeted for HIV prevention and treatment to minimize harm and safeguard users' privacy and confidentiality [53]. Furthermore, it is important that the app be available for users outside of Kuala Lumpur, the capital city, and be linked to both private and government clinics/hospitals. This will ensure widespread implementation of JomPrEP to scale-up HIV prevention services for MSM across Malaysia.

### Limitations

The results of this study should be viewed in the context of the limitations. First, this pilot study included a small sample size and short-term follow-up (ie, 30-days) and used a single-arm design that is commonly used in beta testing; therefore, it was not powered or designed to evaluate efficacy. Second, our participants were subject to selection bias across several dimensions. For example, we recruited men using Facebook or a dating app (ie, Hornet) who may have been more comfortable using mobile apps than other men (ie, hidden MSM). Moreover, participants were already engaged, at least in part, due to their prior high levels of HIV testing. Participants were enrolled in the Greater Kuala Lumpur area only, potentially limiting the generalizability of the findings. Third, social desirability bias may have led participants to speak more positively about their app experience during the survey and exit interviews. This was in part observed by participants who responded favorably to app features, but our usability testing had not confirmed they used the specific feature. Finally, HIVST kits and PrEP services were free to the participants, which may have led to an overestimation of the actual uptake of HIV testing and PrEP services.

### Future Directions

Further research is warranted to examine the implementation of JomPrEP in a more real-world setting. Regardless of these limitations, we believe that our findings carry important implications for efforts to improve the uptake of HIV testing and PrEP services among Malaysian MSM using an app-based intervention.

### Conclusion

Overall, the JomPrEP app represents a feasible and acceptable tool for Malaysian MSM to access HIV prevention services. Importantly, it incorporates several on-demand features to support the remote delivery of HIV prevention services, thus representing a significant innovation on traditional clinic-based and O2O service delivery models [28,29]. The reported outcomes are promising and indicate the benefits of systematically implementing this platform to foster HIV prevention efforts in LMICs, such as Malaysia, where MSM are disenfranchised from existing prevention efforts [2-4]. A large-scale randomized controlled trial is warranted to establish the efficacy of JomPrEP among this at-risk group.

## Appendix

Multimedia Appendix 1 JomPrEP app screenshots.

## Abbreviations

CERiA: Centre of Excellence for Research in AIDS
DAI: discreet app icon
EHR: electronic health record
HIVST: HIV self-testing
JPP: JomPrEP points
LMIC: low- and middle-income country
mHealth: mobile health
MSM: men who have sex with men
O2O: online-to-offline
PEP: postexposure prophylaxis
PrEP: pre-exposure prophylaxis
STI: sexually transmitted infection
SUS: System Usability Scale

## Data Availability

The data sets generated and analyzed during this study are available from the corresponding author on reasonable request.
