# Supplementary material for: Clinic-Integrated Smartphone App (JomPrEP) to Improve Uptake of HIV Testing and Pre-exposure Prophylaxis Among Men Who Have Sex With Men in Malaysia: Mixed Methods Evaluation of Usability and Acceptability
Source: JMIR Mhealth Uhealth. 2023 Feb 16;11:e44468. doi: 10.2196/44468 (PMC9982718; doi:10.2196/44468)
Supplement: Multimedia Appendix 1 [file mhealth_v11i1e44468_app1.pdf]

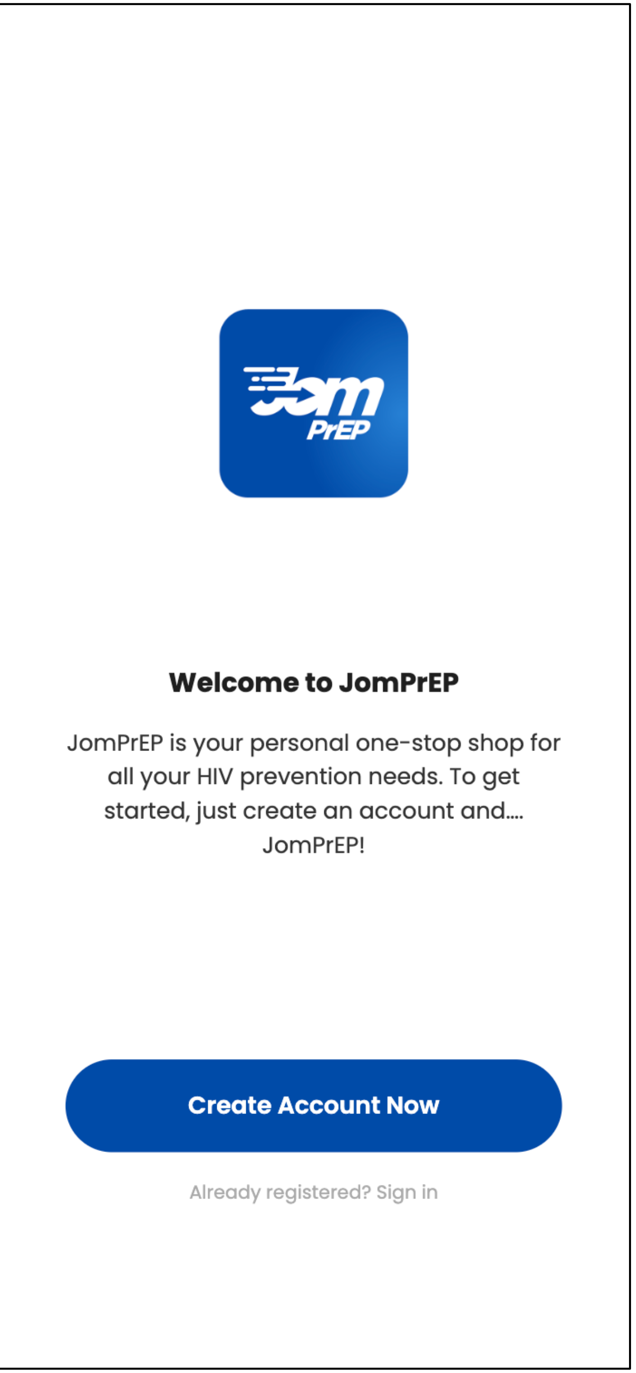
PrEP
Welcome to JomPrEP
JomPrEP is your personal one-stop shop for all your HIV prevention needs. To get started, just create an account and.... JomPrEP!
Create Account Now
Already registered? Sign in

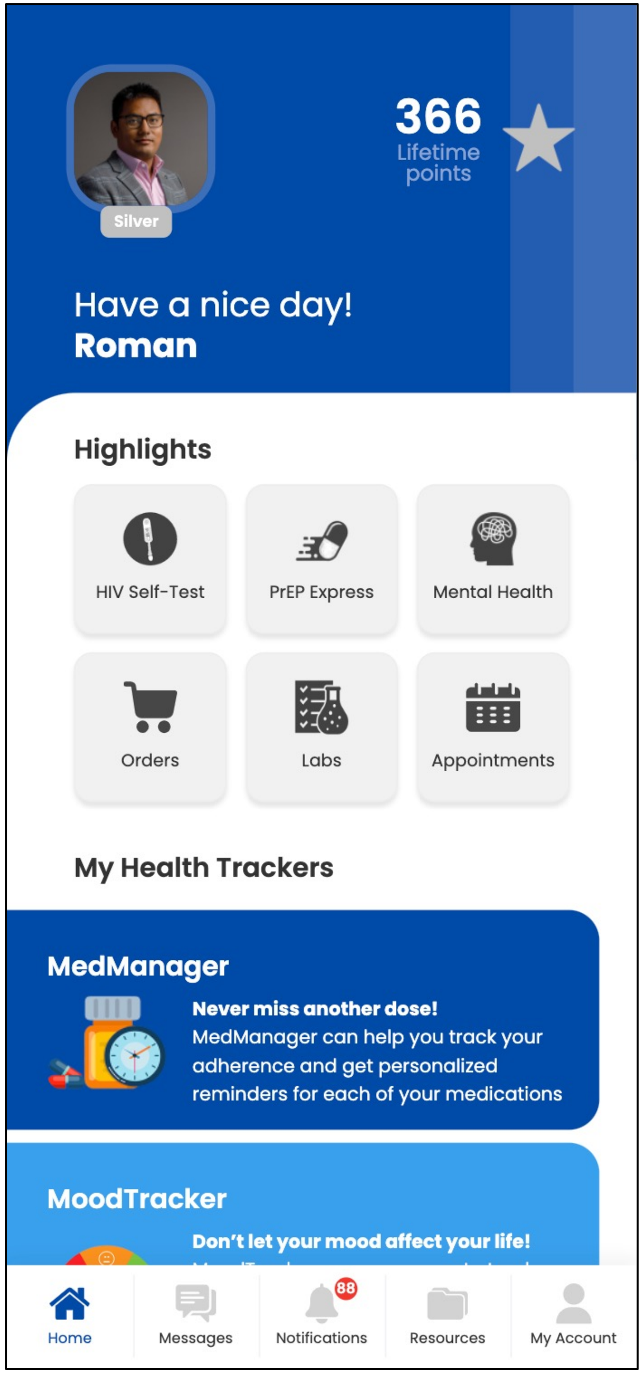
366
Lifetime points
Silver
Have a nice day!
Roman
Highlights
HIV Self-Test
PrEP Express
Mental Health
Orders
Labs
Appointments
My Health Trackers
MedManager
Never miss another dose!
MedManager can help you track your adherence and get personalized reminders for each of your medications
MoodTracker
Don't let your mood affect your life!
88
Home
Messages
Notifications
Resources
My Account

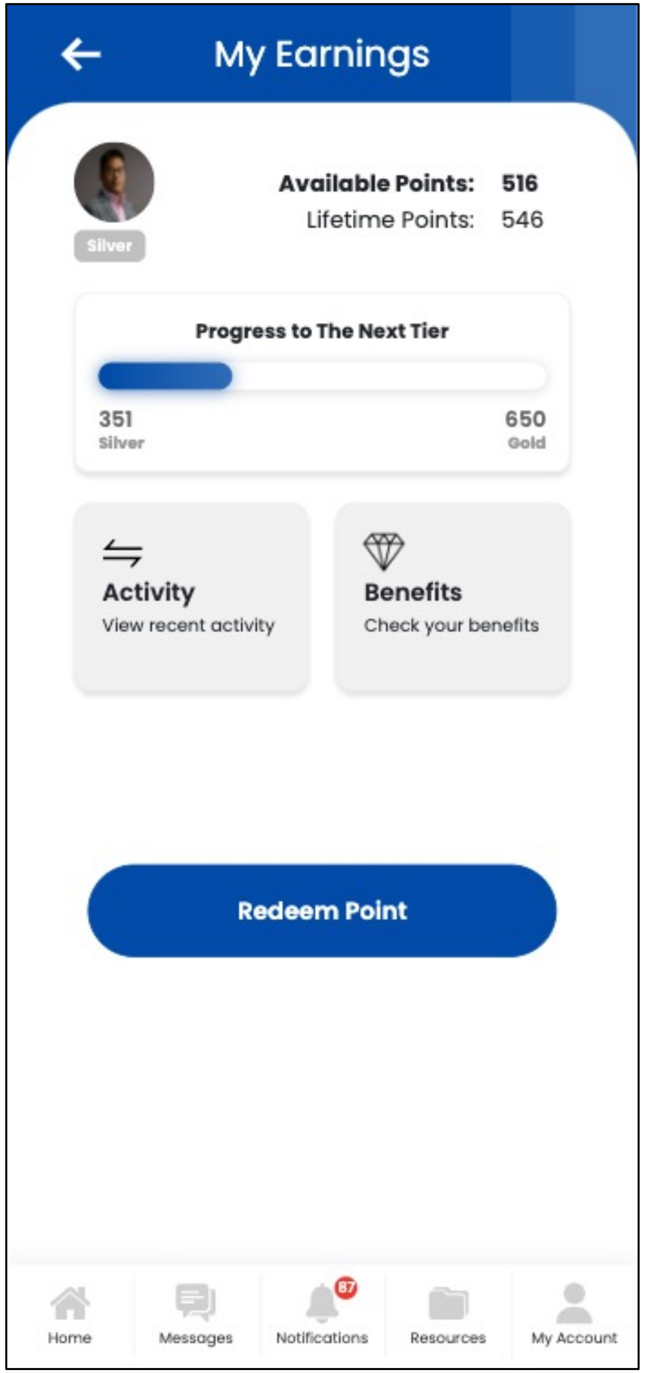
My Earnings
Silver
Available Points: 516
Lifetime Points: 546
Progress to The Next Tier
351
Silver
650
Gold
Activity
View recent activity
Benefits
Check your benefits
Redeem Point
87
Home
Messages
Notifications
Resources
My Account

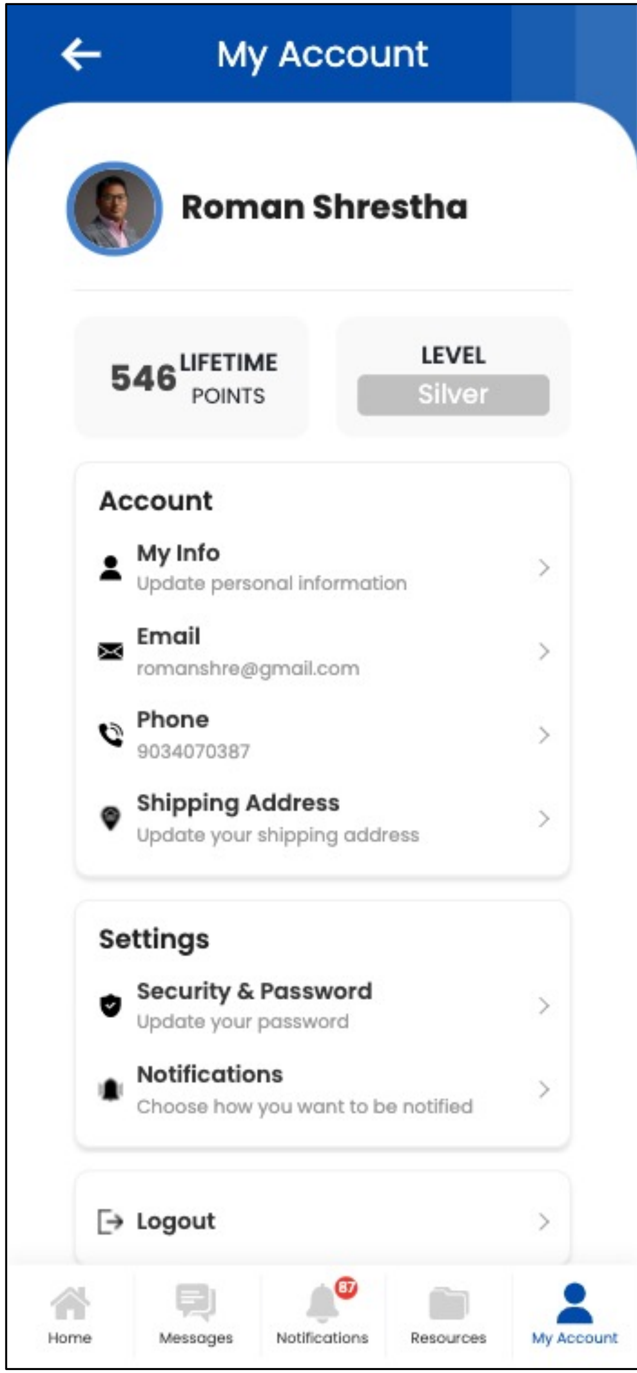
My Account
Roman Shrestha
546 LIFETIME POINTS
LEVEL
Silver
Account
My Info
Update personal information
Email
romanshre@gmail.com
Phone
9034070387
Shipping Address
Update your shipping address
Settings
Security & Password
Update your password
Notifications
Choose how you want to be notified
Logout
87
Home
Messages
Notifications
Resources
My Account

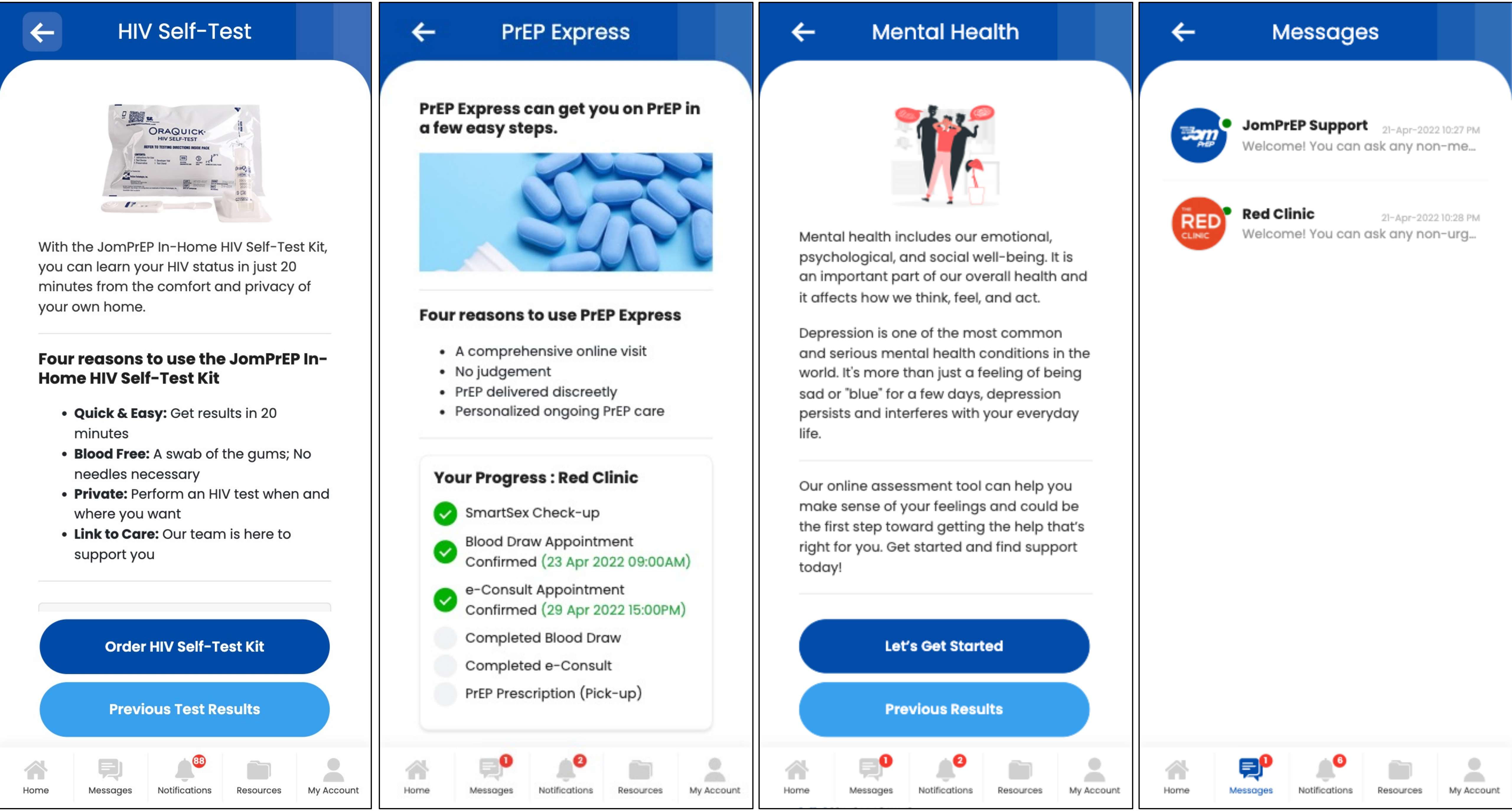
HIV Self-Test
ORAQUICK
HIV SELF-TEST
With the JomPrEP In-Home HIV Self-Test Kit, you can learn your HIV status in just 20 minutes from the comfort and privacy of your own home.
Four reasons to use the JomPrEP In-Home HIV Self-Test Kit
Quick & Easy: Get results in 20 minutes
Blood Free: A swab of the gums; No needles necessary
Private: Perform an HIV test when and where you want
Link to Care: Our team is here to support you
Order HIV Self-Test Kit
Previous Test Results
Home
Messages
88
Notifications
Resources
My Account
PrEP Express
PrEP Express can get you on PrEP in a few easy steps.
Four reasons to use PrEP Express
A comprehensive online visit
No judgement
PrEP delivered discreetly
Personalized ongoing PrEP care
Your Progress : Red Clinic
SmartSex Check-up
Blood Draw Appointment Confirmed (23 Apr 2022 09:00AM)
e-Consult Appointment Confirmed (29 Apr 2022 15:00PM)
Completed Blood Draw
Completed e-Consult
PrEP Prescription (Pick-up)
Home
Messages
1
Notifications
2
Resources
My Account
Mental Health
Mental health includes our emotional, psychological, and social well-being. It is an important part of our overall health and it affects how we think, feel, and act.
Depression is one of the most common and serious mental health conditions in the world. It's more than just a feeling of being sad or "blue" for a few days, depression persists and interferes with your everyday life.
Our online assessment tool can help you make sense of your feelings and could be the first step toward getting the help that's right for you. Get started and find support today!
Let's Get Started
Previous Results
Home
Messages
1
Notifications
2
Resources
My Account
Messages
JomPrEP Support
21-Apr-2022 10:27 PM
Welcome! You can ask any non-me...
Red Clinic
21-Apr-2022 10:28 PM
Welcome! You can ask any non-urg...
RED
CLINIC
Home
Messages
1
Notifications
6
Resources
My Account

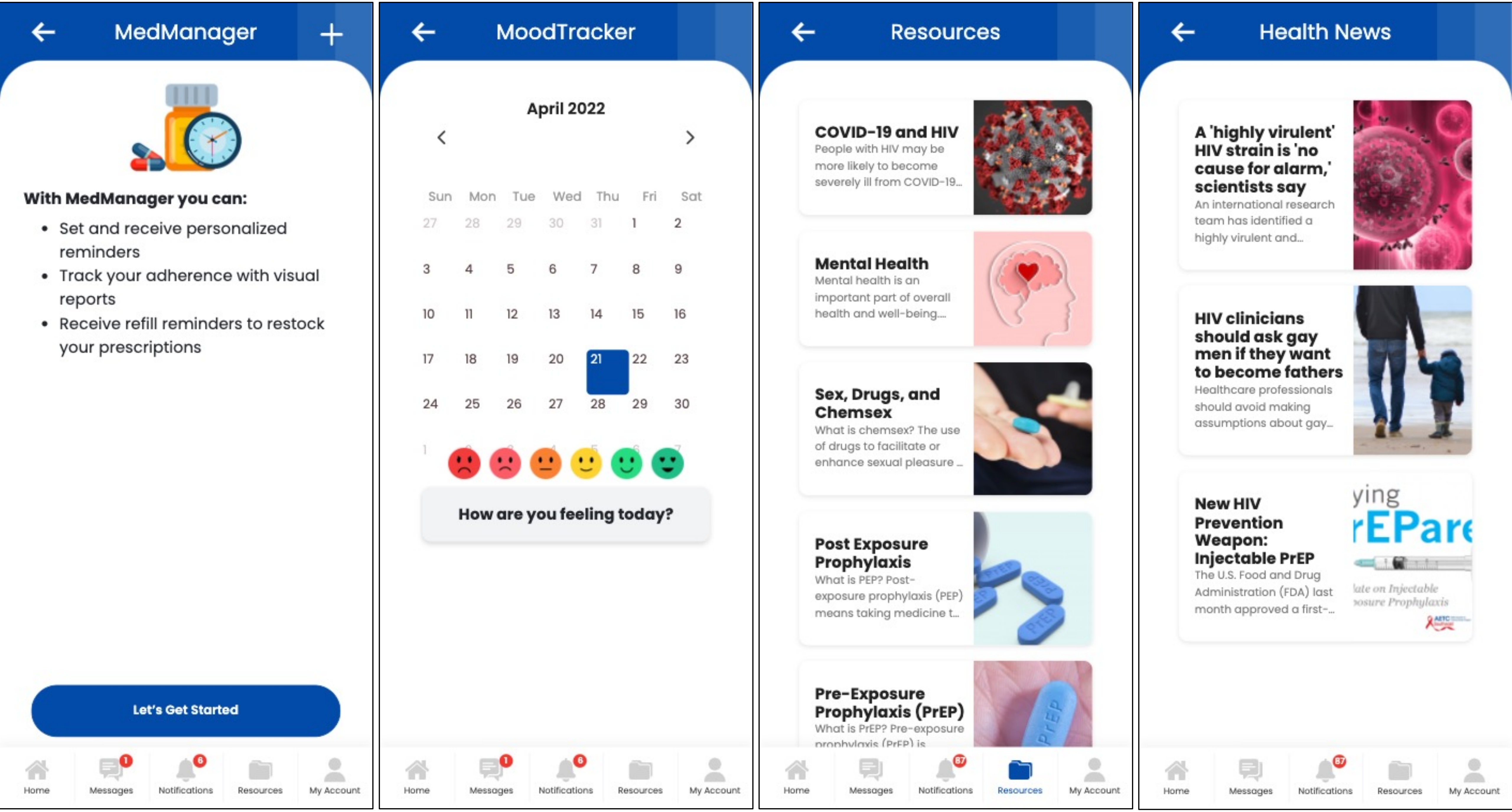
MedManager
With MedManager you can:
Set and receive personalized reminders
Track your adherence with visual reports
Receive refill reminders to restock your prescriptions
Let's Get Started
Home
Messages
Notifications
Resources
My Account
MoodTracker
April 2022
Sun Mon Tue Wed Thu Fri Sat
27 28 29 30 31 1 2
3 4 5 6 7 8 9
10 11 12 13 14 15 16
17 18 19 20 21 22 23
24 25 26 27 28 29 30
How are you feeling today?
Resources
COVID-19 and HIV
People with HIV may be more likely to become severely ill from COVID-19...
Mental Health
Mental health is an important part of overall health and well-being....
Sex, Drugs, and Chemsex
What is chemsex? The use of drugs to facilitate or enhance sexual pleasure ...
Post Exposure Prophylaxis
What is PEP? Post-exposure prophylaxis (PEP) means taking medicine t...
Pre-Exposure Prophylaxis (PrEP)
What is PrEP? Pre-exposure
Health News
A 'highly virulent' HIV strain is 'no cause for alarm,' scientists say
An international research team has identified a highly virulent and...
HIV clinicians should ask gay men if they want to become fathers
Healthcare professionals should avoid making assumptions about gay...
New HIV Prevention Weapon: Injectable PrEP
The U.S. Food and Drug Administration (FDA) last month approved a first-...
